# Real-Time Hybrid Test Control Research Based on Improved Electro-Hydraulic Servo Displacement Algorithm

**DOI:** 10.3390/s23104765

**Published:** 2023-05-15

**Authors:** Yaoyu Shen, Ying-Qing Guo, Xiumei Zha, Yina Wang

**Affiliations:** College of Mechanical and Electronic Engineering, Nanjing Forestry University, Nanjing 210037, China; shenyaoyu@njfu.edu.cn (Y.S.); zhaxiumei@njfu.edu.cn (X.Z.); wangyina@njfu.edu.cn (Y.W.)

**Keywords:** real-time hybrid test, electro-hydraulic servo, FF-PSO-PID, composite control, PSO algorithm

## Abstract

Real-time hybrid testing (RTH) is a test method for dynamic loading performance evaluation of structures, which is divided into digital simulation and physical testing, but the integration of the two may lead to problems such as time lag, large errors, and slow response time. The electro-hydraulic servo displacement system, as the transmission system of the physical test structure, directly affects the operational performance of RTH. Improving the performance of the electro-hydraulic servo displacement control system has become the key to solving the problem of RTH. In this paper, the FF-PSO-PID algorithm is proposed to control the electro-hydraulic servo system in real-time hybrid testing (RTH), which uses the PSO algorithm to operate the optimized PID parameters and the feed-forward compensation algorithm to compensate the displacement. First, the mathematical model of the electro-hydraulic displacement servo system in RTH is presented and the actual parameters are determined. Then, the objective evaluation function of the PSO algorithm is proposed to optimize the PID parameters in the context of RTH operation, and a displacement feed-forward compensation algorithm is added for theoretical study. To verify the effectiveness of the method, joint simulations were performed in Matlab/Simulink to compare and test FF-PSO-PID, PSO-PID, and conventional PID (PID) under different input signals. The results show that the proposed FF-PSO-PID algorithm effectively improves the accuracy and response speed of the electro-hydraulic servo displacement system and solves the problems of RTH time lag, large error, and slow response.

## 1. Introduction

The real-time hybrid test (RTH) [1] is a test method to study the performance of testing the response of large structures under dynamic loading. The whole test structure consists of a numerical substructure and a physical substructure, the former is calculated by a computer for numerical simulation, and the latter act as the displacement on the physical experimental structure by communicating with the electro-hydraulic servo displacement system through the computer [2,3]. As a test system with high requirements for real-time, RTH, during the numerical simulation calculation, signal output, and signal acquisition, the hydraulic cylinder actuator detects the displacement and the desired displacement. There are problems with hysteresis and errors, and the electro-hydraulic servo system, as an actuator, affects the performance of the whole RTH system.

The electro-hydraulic servo system [4,5], as a subsystem of the real-time hybrid test, has high intrinsic frequency and loop gain, high control accuracy of displacement, velocity, force, and excellent tracking performance. In recent years, research on electro-hydraulic servo control systems has focused on the online identification of nonlinear parameters [6,7,8,9,10], state observation [11,12,13,14,15,16], fault detection [17], dead zone compensation [18], PID algorithms [19], sliding mode control [20], neural networks [21,22,23], and other methods to achieve accurate control of the system.

In the development of the real-time hybrid test, Nakashima et al., 1992; Horiuchi et al., 1999; Horiuchi et al., 2001 [24,25,26] found that the hysteresis and errors of the electro-hydraulic servo actuator directly affect the test results of the real-time hybrid test in the experiment. In order to achieve the test results, control algorithms must be introduced to improve the system’s performance. With the development of electro-hydraulic servo systems in the past 20 years, the research of electro-hydraulic control algorithms has been continuously advanced, and sliding mode control [27,28], robust control [29], and PID control [30] have become the main research content of scholars. In comparison, PID control algorithms are widely used in electro-hydraulic servo system control. With the development of artificial intelligence science, the combination of artificial intelligence algorithms [31,32,33] and PID control algorithms has become the mainstream trend and Hao et al. [34] proposed an improved particle swarm optimization for the trajectory tracking accuracy problem to optimize the proportional-integral differential (PID) controller coefficients of the PSO algorithm, which obtained high accuracy and fast convergence of the electro-hydraulic servo system; nevertheless, the problem of system lag and slow response exists. Fan [35] and Ma et al. [36] improved the control performance by improving the objective evaluation function of the PSO algorithm, but the proposed objective evaluation function is too simple for the system performance index requirements, which may be ineffective or inefficient for processing complex signals, and has not been validated and tested in various situations. Guo et al. [37] proposed a new objective evaluation function in genetic algorithm rectified PID with a penalty mechanism. However, excessive pursuit of response rate leads to oscillation in the early stage, and the introduction of other control algorithms combined with PID control is worthy of reference.

From the above work, it can be seen that scholars have extensively used the PSO algorithm to modify the PID parameters and improve the PSO algorithm from different perspectives to improve its performance. The main focus has been on modifying the objective evaluation function of the PSO algorithm, introducing other control algorithms, and resetting the parameters of the algorithm. However, none of them effectively solves the system response lag problem, and the application environment of the algorithm is not related to real-time hybrid testing (RTH) and performs poorly under complex input signals. The main contribution of this paper is to propose the FF-PSO-PID algorithm to optimize the electro-hydraulic servo system in real-time hybrid testing (RTH), which uses the PSO algorithm to optimize the PID parameters and the feed-forward compensation algorithm to compensate the displacement, and the FF-PSO-PID algorithm is a combination of two different algorithms. In this paper, the objective evaluation function of the PSO algorithm is proposed, and a penalty mechanism considering the system error and the system response speed is added. Based on the optimized PID of PSO algorithm, a feed-forward compensation algorithm is introduced and combined with PSO-PID algorithm for hybrid control, which improves the system response speed, convergence speed, and tracking performance and effectively solves the problem of system response lag. In this paper, the algorithm is applied to various complex input signals to verify its excellent performance, and it is also verified that the algorithm meets the performance requirements of real-time hybrid testing (RTH).

## 2. Real-Time Hybrid Test System

**Figure 1:**
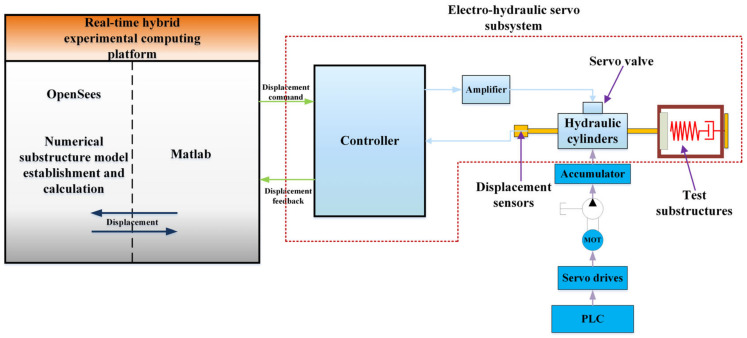
Real-time hybrid test system.

**Figure 2:**
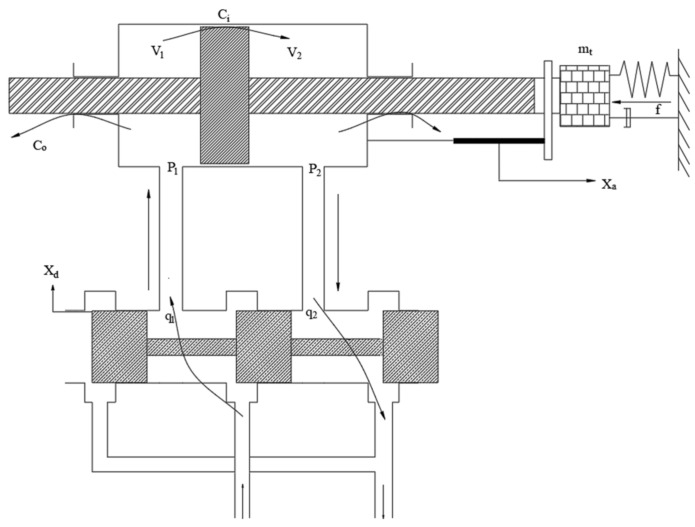
Hydraulic cylinder model.

In this paper, as shown in Figure 1, the electro-hydraulic servo system, as a subsystem of the hybrid test system, is the most important part in the context of the hybrid test. This paper focuses on the electro-hydraulic servo system as the research object to improve the performance of the whole real-time hybrid test (RTH) by improving the control algorithm of the electro-hydraulic servo system. The electro-hydraulic servo system is mainly composed of eight parts, such as controller, servo valve, hydraulic cylinder and displacement sensor. As shown in Figure 2, the main working principle is to send a voltage signal from the controller, through the amplifier into current, the size of the valve port of the electro-hydraulic servo valve to control, so as to determine the flow direction and flow of oil into the hydraulic cylinder, the oil in the hydraulic cylinder to push the actuator piston rod, and the load for movement. The displacement sensor at the actuator end feeds back the displacement signal to form the closed-loop control of the electro-hydraulic servo system.

### 2.1. Electro-Hydraulic Servo System

#### 2.1.1. Hydraulic Cylinder Model

Using the four-sided slide valve as a prototype, the four-way valve load flow equation is linearizable, as shown in Figure 2.

According to the physical properties of the four-sided slide valve and the literature proof [37], the hydraulic cylinder can be approximated as a third-order oscillating system. The formula is as follows:

(1) $$\begin{array}{l} G_{B}(s)=\frac{X_{a}}{X_{d}}=\frac{\frac{K_{q}}{A_{p}}}{\frac{m_{t}V_{t}}{4\beta_{e}A_{p}^{2}}s^{3}+(\frac{m_{t}K_{\mathrm{ce}}}{A_{P}^{2}}+\frac{B_{p}V_{t}}{4\beta_{e}A_{p}^{2}})s^{2}+s}\\ =\frac{1/A_{p}}{s(\frac{s^{2}}{\omega_{r}^{2}}+\frac{2\xi_{r}}{\omega_{r}}s+1)} \end{array}$$

where $\omega_{r}$ = $\sqrt{\frac{4\beta_{e}A_{p}^{2}}{m_{t}V_{t}}}$, $\omega_{r}$ is the hydraulic inherent frequency; as $B_{p}$ is small and negligible, it is simplified to $\delta_{r}=\frac{K_{\mathrm{ce}}}{A_{p}}\sqrt{\frac{m_{t}}{V_{t}}}$, which is the hydraulic damping ratio.

#### 2.1.2. Servo Valve Model

Using the G761 series electro-hydraulic servo valve made by Moog as a prototype, the servo valve can be approximated as a second-order oscillating system when the servo valve bandwidth is close to the hydraulic inherent frequency [37].

(2) $$G_{A}(s)=\frac{X_{v}(s)}{I_{c}(s)}=\frac{\frac{k_{c}k_{\mathrm{qx}}A}{k_{\mathrm{qp}}k}}{\frac{m}{k}s^{2}+(\frac{f}{k}+\frac{A^{2}}{k_{\mathrm{qp}}k})s+1}=\frac{K_{\mathrm{sv}}}{\frac{1}{\omega_{n}^{2}}s^{2}+2\frac{\xi_{n}}{\omega_{n}}s+1}$$

where $K_{\mathrm{sv}}$ is the electro-hydraulic servo valve proportional gain; $\omega_{n}$ is the intrinsic frequency; $\xi_{n}$ is the damping ratio.

#### 2.1.3. Servo Amplifier and Displacement Sensor Model

The servo amplifier can be simplified to a proportional link, and we let this proportional link be $K_{a}$. The input displacement signal is the voltage value, the range is ±10 V, and the input current range of the electro-hydraulic servo valve is 0–±40 mA.

The feedback signal is the displacement signal. Similarly, the displacement range is 220 mm, the input is voltage ±10 V, and we set the feedback amplifier as $K_{s}$.

## 3. PID Control Algorithm

A controller with a proportional-integral-derivative control law is called a PID controller.

This combination has the characteristics of each of the three basic laws, and its equation of motion is:

(3) $$m(t)=K_{p}e(t)+\frac{K_{i}}{T_{i}}\int_{0}^{t}e(t)\mathrm{dt}+K_{d}\tau \frac{\mathrm{de}(t)}{\mathrm{dt}}$$

The corresponding transfer function is:

(4) $$G_{\mathrm{pid}}(s)=(K_{p}+\frac{K_{i}}{s}+K_{d}s)E(s)$$

**Figure 3:**
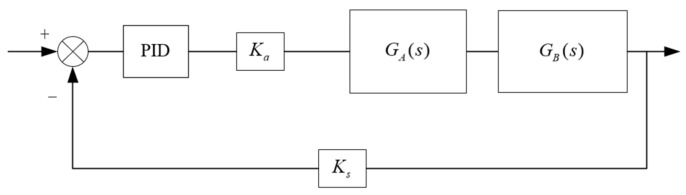
Electro-hydraulic servo system PID control.

As shown in Figure 3, the PID algorithm can effectively perform closed-loop control of the electro-hydraulic servo system for real-time hybrid test, but the three parameters are very difficult to determine, and it is difficult to find the specific parameters accurately, causing the control system to fail to operate efficiently.

## 4. FF-PSO-PID Control Algorithm

The algorithm in this paper is a combination of PSO-optimized PID parameters and a feed-forward compensation algorithm (FF-PSO-PID). While improving the tracking error and response rate of the electro-hydraulic servo system for real-time hybrid testing, the system response hysteresis is further addressed.

### 4.1. PSO Algorithm Optimizes PID Parameters

In this paper, the objective evaluation function of the PSO algorithm is improved to accurately calculate the three parameters of the PID applicable to the real-time hybrid test to ensure that the system error, response speed, and tracking performance meet the test requirements.

#### 4.1.1. PSO Update Rules

The PSO is initialized as a group of random particles (random solutions). The optimal solution is found by iteration.

The particle updates its velocity and position by the following equation [36]:

(5) $$v_{i}=v_{i}+c_{1}\times \mathrm{rand}()\times ({\mathrm{pbest}}_{i}-x_{i})+c_{2}\times \mathrm{rand}()\times ({\mathrm{gbest}}_{i}-x_{i})$$

(6) $$x_{i}=x_{i}+v_{i}$$

In Equation (5), i=1,2,⋯,N,N is the total number of particles in the population. $v_{i}$ is the velocity of the particle, rand() is a random number between (0,1), and $c_{1}$ and $c_{2}$ are learning factors.

Introducing the inertia weighting factor ω based on the standard form of the above base PSO, the following equation is obtained:

(7) $$v_{i}=\omega \times v_{i}+c_{1}\times \mathrm{rand}()\times ({\mathrm{pbest}}_{i}-x_{i})+c_{2}\times \mathrm{rand}()\times ({\mathrm{gbest}}_{i}-x_{i})$$

where ω is the inertia weight factor, the magnitude of its value affects the ability of global search and local search, and a dynamic ω can obtain better search results.

#### 4.1.2. Design of the Objective Evaluation Function

According to the characteristics of electro-hydraulic servo displacement control, the following objective evaluation function is proposed:

(8) $$\mathrm{Min}[J=\int_{0}^{\infty }(\omega_{1}t\left|e(t)\right|+\omega_{2}t_{r})\mathrm{dt}+\omega_{3}t_{s}+\omega_{4}\sigma \%]$$

where $\omega_{1}$, $\omega_{2}$, $\omega_{3},$ and $\omega_{4}$ are the weights of the performance indicators, and different weights affect different system performance effects. σ% is the overshoot, $\int_{0}^{\infty }t\left|e(t)\right|\mathrm{dt}$ is the time integral of the absolute value of the error multiplied by time, $t_{s}$ is the regulation time, and $t_{r}$ is the rise time. A penalty factor is placed in the objective function J, and when the regulation time is greater than 0.3, it is no longer calculated. When overshoot occurs, the amount of overshoot is used as one of the optimal indicators.

In the simulation test process, $\omega_{1}$, $\omega_{2}$, $\omega_{3}$ and $\omega_{4}$ in equations are 0.8, 0.3, 0.7, and 0.5, respectively, to take into account both the ability of the system to respond quickly, to be accurate without errors, to have good tracking performance, and to converge quickly. In this engineering test, the smaller the value of the target value J, the better the performance effect of the system is indicated.

### 4.2. Feed-Forward Compensation Control

In order to achieve direct control without hysteresis, improve the response rate of the system, obtain the corresponding increased accuracy to know the controlled object model and system characteristics, the introduction of feed-forward control on the basis of PID control. Feedback control requires deviations to occur before regulation, with hysteresis.

**Figure 4:**
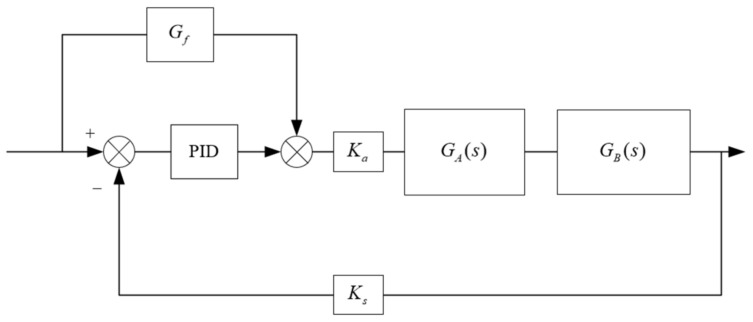
Feed-forward displacement compensation system.

A compound control system with input compensation is shown in Figure 4. The $G_{f}(s)$ in Figure 4 is the transfer function of feed-forward compensation, $G_{A}(s)$ and $G_{B}(s)$ are the control objects of servo valve and hydraulic cylinder respectively, and $K_{a}$ and $K_{s}$ are the servo amplifiers.

From the figure, it can be seen that the system output is:

(9) $$C(s)=K_{a}K_{s}G_{A}(s)G_{B}(s)\left[E(s)G_{\mathrm{pid}}(s)+G_{f}(s)R(s)\right]$$

The errors caused by the system are as follows:

(10) E(s)=R(s)−C(s)

The following can be obtained:

(11) $$C(s)=\frac{K_{a}K_{s}G_{A}(s)G_{B}(s)\left[G_{\mathrm{pid}}(s)+G_{f}(s)\right]}{1+K_{a}K_{s}G_{A}(s)G_{B}(s)G_{\mathrm{pid}}(s)}R(s)$$

In order to make the system error zero, $E\left(s\right)=0$.

Suppose $E\left(s\right)=R(s)\neq 0$, substitution into Equations (10) and (11) yields

(12) $$G_{f}(s)=\frac{A_{p}}{K_{a}K_{s}K_{\mathrm{sv}}}(a_{1}s^{5}+a_{2}s^{4}+a_{3}s^{3}+a_{4}s^{2}+a^{5}s)$$

in the formula

$$\begin{array}{l} a_{1}=\frac{1}{\omega_{n}^{2}\omega_{r}^{2}},\;a_{2}=2\frac{\xi_{n}\omega_{n}+\xi_{r}\omega_{r}}{\omega_{n}^{2}\omega_{r}^{2}}\\ a_{3}=\frac{\omega_{n}^{2}+4\omega_{n}\omega_{r}\xi_{n}\xi_{r}+\omega_{r}^{2}}{\omega_{n}^{2}\omega_{r}^{2}},\;a_{4}=2\frac{\omega_{n}\xi_{r}+\omega_{r}\xi_{n}}{\omega_{r}\omega_{n}}\\ a_{5}=1 \end{array}$$

The sixth-order system greatly increases the hardware requirements and is not easily implemented at a high cost. Thus, here, the simplification process is performed and the second-order term is retained to achieve compensation for the system.

Therefore, the feed-forward compensation function after simplification is

(13) $$G_{f}(s)=\frac{A_{p}}{K_{a}K_{s}K_{\mathrm{sv}}}(2\frac{\omega_{n}\xi_{r}+\omega_{r}\xi_{n}}{\omega_{r}\omega_{n}}s^{2}+s)$$

Simplifying from the data in Table 1 yields

(14) $$G_{f}(s)=0.002s^{2}+0.6405s$$

### 4.3. Hybrid Algorithm for Feed-Forward Compensation and Improved PSO-Optimized PID

**Figure 5:**
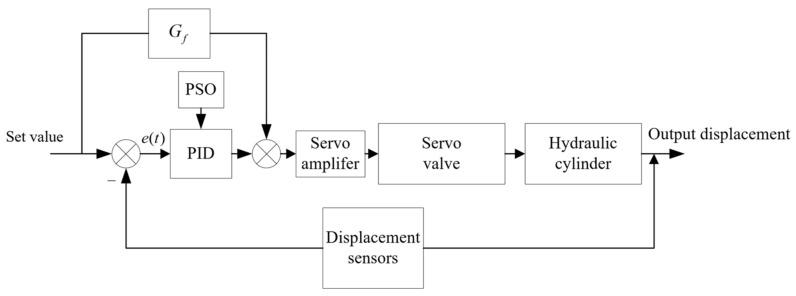
FF-PSO-PID algorithm control system.

In this paper, the FF-PSO-PID algorithm is proposed to control the electro-hydraulic servo system in the real-time hybrid test (RTH), which uses the PSO algorithm to optimize the PID parameters and the feed-forward compensation algorithm to compensate the displacement. As shown in Figure 5, on the basis of the PSO algorithm, to rectify the PID parameters and reduce the system displacement error and response time, feed-forward displacement compensation is introduced to reduce the system error through feed-forward compensation of the direct control system, while improving the response speed and reducing the unnecessary system lag phenomenon.

## 5. Simulation and Analysis

In this section, the performance of the conventional PID algorithm, PSO-PID, and FF-PSO-PID are compared through three different displacement input signals with the control objective of making the electro-hydraulic servo system respond quickly and track the reference trajectory with satisfactory tracking accuracy.

**Figure 6:**
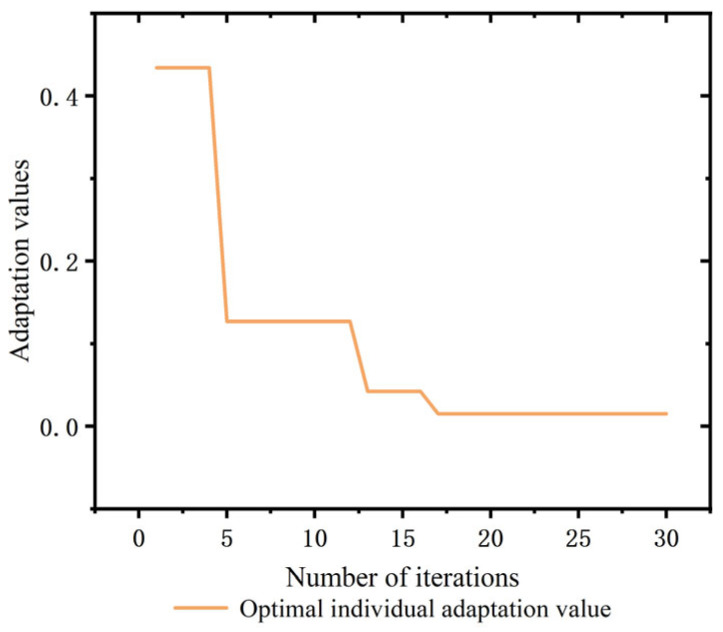
Adaptive degree value of the PSO algorithm.

The electro-hydraulic servo control model is established in Matlab/Simulink with the physical models of servo valve $G_{A}$, hydraulic cylinder $G_{B}$ and servo amplifiers $K_{a}$ and $K_{s}$ as transfer functions, and the models of servo valve and hydraulic cylinder are encapsulated using Matlab function module, and the specific parameters are shown in Table 1. The parameters of the PSO algorithm are shown in Table 2, and its adaptive degree value is shown in Figure 6, when the optimal value is reached at the 16th iteration, the value is 0.0149. The parameters of the conventional PID and PSO-PID are shown in Table 3. 

**Figure 7:**
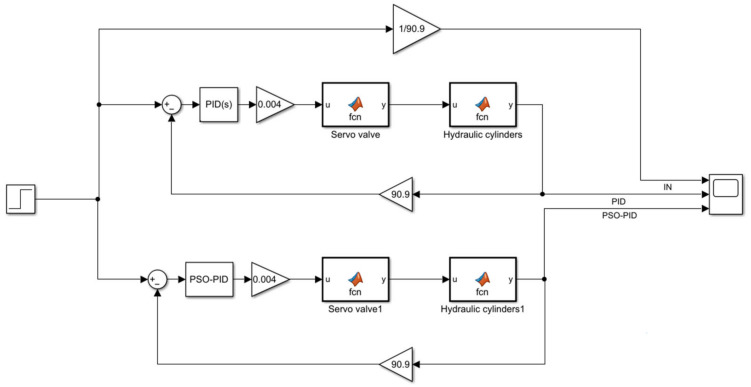
Simulation diagram of PID and PSO-PID.

**Figure 8:**
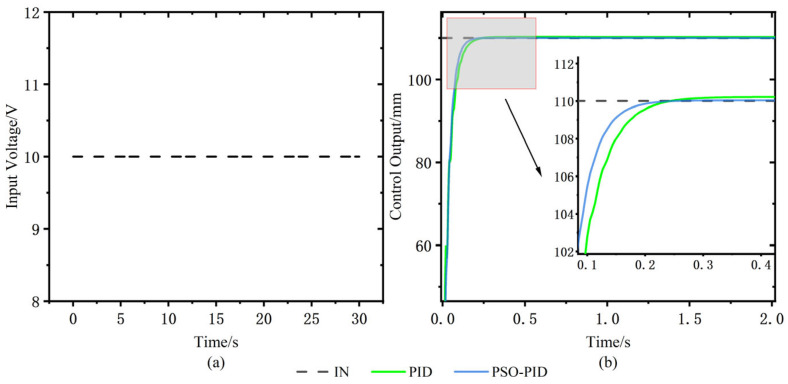
(**a**) Step input signal; (**b**) Conventional PID and PSO-PID response curves under step signal.

**Figure 9:**
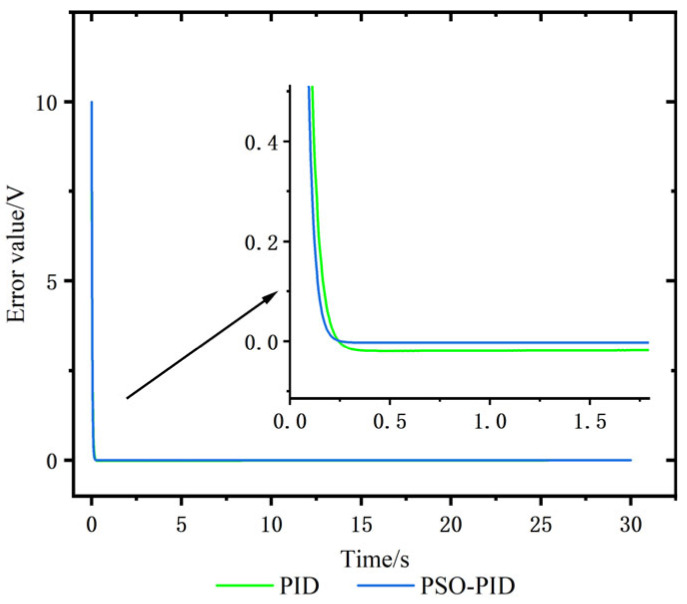
Error response curve under step signal.

A step signal of 110.011 mm is used as the reference trajectory of the electro-hydraulic displacement servo system, and the conventional PID and PSO-PID are compared and analyzed. Modeled in Simulink, the simulation plots of the two control algorithms are shown in Figure 7, Figure 8a shows the input voltage, and the response curves under the step signal are shown in Figure 8b. The error response curves of the two algorithms under the step signal are shown in Figure 9.

**Figure 10:**
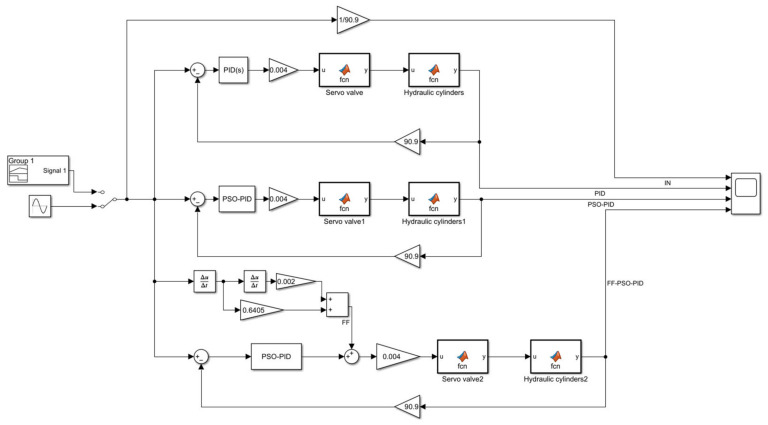
Simulation of conventional PID, PSO-PID, and FF-PSO-PID with sinusoidal and random signals.

From the comparison results in Table 4 and Figure 8 and Figure 9, it can be seen that the response performance of the conventional PID algorithm is poor. The maximum response of the conventional PID control curve is 110.224 mm, the peak time is 0.46 s, and the overshoot is 0.2%, while the maximum response of the PSO-PID control curve is 110.039 mm, the peak time is 0.36 s, and the overshoot is 0.036%, which is 0.164% lower than that of the conventional PID. The regulation time $t_{s}$ of conventional PID is 0.109 s, and the $t_{s}$ of PSO-PID is 0.093 s, which is 0.016 s lower than that of conventional PID, and PSO-PID has to enter the steady-state value earlier than conventional PID. Taking the rise time of the output response from 10% of the target value to 90% of the target value, the rise time $t_{r}$ of the common PID control curve is 0.007 s, while the rise time $t_{r}$ of the PSO-PID control curve is 0.006 s, which shows that the response of PSO-PID is faster than that of the conventional PID. As shown in Figure 10, the error between PSO-PID and the target value is close to 0 after 0.2 s, while the conventional PID takes 0.246 s and has a larger error with the target value. The overshoot of PSO-PID is reduced by 0.164% compared with the conventional PID algorithm, and the peak time is only 0.36 s. The steady-state displacement error of PSO-PID algorithm is reduced by 0.174 mm compared with the conventional PID algorithm. Therefore, it is verified that the performance of PSO-PID is better than the conventional PID and effectively reduces the displacement error, but the improvement in response speed is smaller, as seen by the rise time and peak time performance.

The conventional PID, PSO-PID, and FF-PSO-PID are compared and analyzed under sinusoidal and random signals.

**Figure 11:**
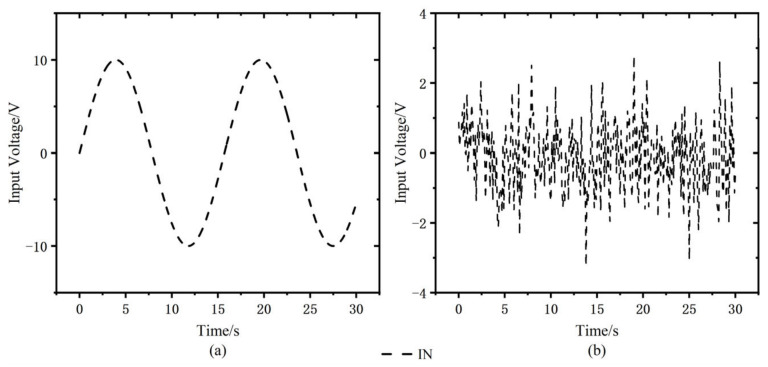
(**a**) Sine input signal; (**b**) Random input signal.

**Figure 12:**
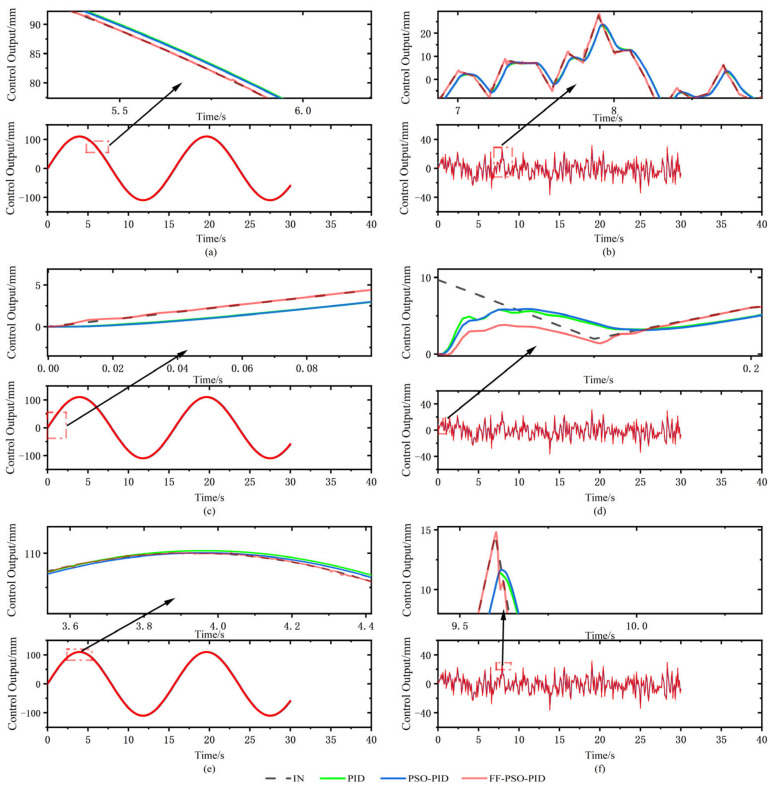
Response curves under sinusoidal and random signals. (**a**) local enlargement of sine signal; (**b**) local enlargement of random signal; (**c**) enlargement of starting position of sine signal; (**d**) enlargement of starting position of random signal; (**e**) enlargement of wave crest of sine signal; (**f**) enlargement of wave crest of random signal.

A sinusoidal signal with an amplitude of 110.011 mm and a frequency of 0.4 Hz is used as the reference trajectory of the electro-hydraulic displacement servo system, which is modeled in Simulink, and the simulation plots of the three algorithms are shown in Figure 10. The input voltage is shown in Figure 11a for a sinusoidal signal with a voltage value of 10 V, which can be converted by the amplifier to obtain a sine wave with an amplitude of 110.011 mm, and the sinusoidal response curves of the three algorithms are shown in Figure 12a,c,e.

From the response curves, there were significant differences among the three algorithms, with the largest error occurring at the same position of the sinusoidal signal, at the crest or trough, when the hydraulic cylinder was in the reversing phase. Among the three controllers, the maximum error of the traditional PID algorithm is 0.177 mm, and the response is the slowest with a lag of 0.04 s. The error of the PSO-PID algorithm is smaller than that of the traditional PID, and the displacement error is reduced by 0.15 mm. The maximum error and lag time of the FF-PSO-PID algorithm are approximately zero, which is the best performance among the three algorithms, verifying that the FF-PSO-PID algorithm is able to meet the requirements of the control after the feed-forward compensation. It is verified that the FF-PSO-PID algorithm can meet the control requirements after the feed-forward compensation effect.

The stochastic signal is used as the reference trajectory of the electro-hydraulic displacement servo system, and the simulation plots of the three algorithms are shown in Figure 10. Figure 11b shows the input voltage signal, and the random signal response curves of the three algorithms are shown in Figure 12b,d,f.

From the three tracking curves, the FF-PSO-PID algorithm has the fastest response and the highest tracking accuracy. Since the simulation is a random signal, considering the response time, the response delay of FF-PSO-PID algorithm is 0.001 s, while the response time of traditional PID and PSO-PID algorithms are 0.027 s and 0.026 s, respectively. FF-PSO-PID improves the response time by 96.3% and 96.2% compared with the other two algorithms. Considering the maximum tracking error, the FF-PSO-PID algorithm is only 0.1 mm, while the conventional PID and PSO-PID algorithms are 4.456 mm and 3.866 mm, which cannot achieve the tracking accuracy required by the system. The average error of the FF-PSO-PID algorithm is 0.08 mm at the peak or trough of the random signal, while the average errors of the conventional PID and PSO-PID algorithms are 3.967 mm and 3.596 mm. In terms of displacement error, FF-PSO-PID improved by 97.75% and 97.41% compared with the other two algorithms. The superiority of the FF-PSO-PID algorithm was verified, which effectively improved the system tracking performance and reduced the displacement error and response time. The FF-PSO-PID algorithm solves the problem of system response hysteresis.

**Figure 13:**
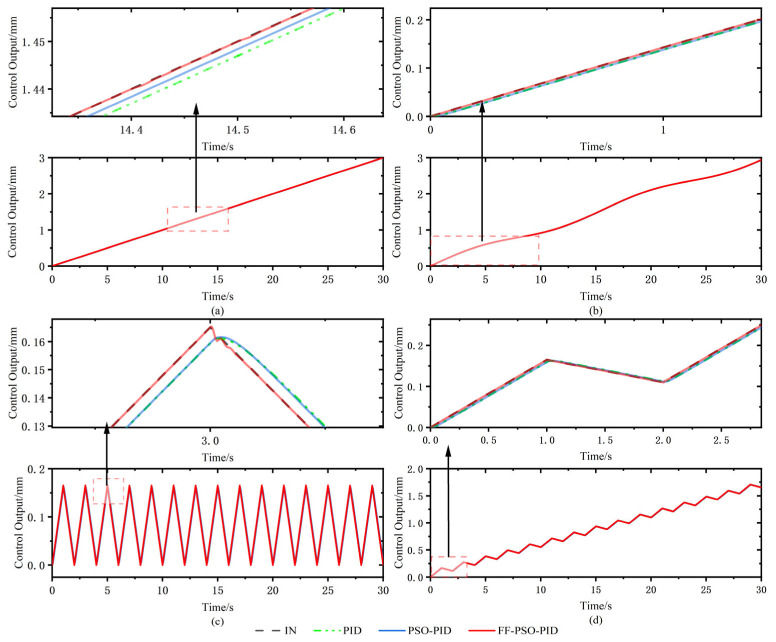
Response curves under different signals. (**a**) local enlargement of the ramp signal; (**b**) local enlargement of the ramp sine synthesis signal; (**c**) local enlargement of the triangular wave signal; (**d**) enlargement of the start position of the triangular wave ramp synthesis signal.

In order to verify the applicability and effectiveness of the algorithm for different input signals, based on the verification by step signal, sinusoidal signal, and random signal, simulation tests were conducted under the signals such as ramp and triangle wave as in Figure 13, respectively. As shown in Figure 13a, the input signal is a ramp signal with a slope of 0.1, and the tracking error and time hysteresis of the FF-PSO-PID algorithm are close to 0, while the errors of PSO-PID and conventional PID are 0.002 mm and 0.004 mm, and the hysteresis times are 0.024 s and 0.05 s, respectively. As shown in Figure 13c, at the crest of the wave under the triangular wave signal, the tracking error and time lag of FF-PSO-PID algorithm are close to 0. The error and time lag of PSO-PID and conventional PID algorithms are more obvious, the displacement error reaches 0.01 mm and 0.009 mm, and the time lag is 0.028 s and 0.031 s, respectively. In comparison, FF-PSO-PID algorithm has applicability and effectiveness for various input signals. The tracking performance is superior to that of the other two algorithms.

## 6. Conclusions

In this paper, to improve the response speed and tracking performance of the real-time hybrid test (RTH) system and to solve the problems of test system response lag and complex input signals, a mathematical model of the servo valve-controlled hydraulic cylinder is developed for the nonlinearity and uncertainty of the electro-hydraulic servo system in the real-time hybrid test (RTH). The FF-PSO-PID algorithm is proposed and simulated under a variety of input signals. The results of the study show that:

(1) The conventional PID relies on manual experience requiring a lot of time for debugging, it cannot find the appropriate parameters accurately, and it has the disadvantages of large displacement error and long response time. Therefore, for the application background of a real-time hybrid test, the control algorithm of PSO-PID for improving electro-hydraulic servo system is proposed accordingly. In the experiments, the PSO-PID algorithm effectively reduces the displacement error, but the response speed is not improved much, and the hysteresis problem still exists.
(2) In order to improve the tracking performance and response speed of the real-time hybrid test (RTH) system and solve the problems of the system in response lag and complex input signal, displacement feed-forward compensation control is introduced based on the PSO-PID algorithm. Through experimental comparison, the proposed FF-PSO-PID algorithm effectively improves the response speed of the electro-hydraulic servo system, reduces the displacement error, and solves the problem of system response hysteresis. The superior tracking accuracy is obtained under multiple input signals, verifying that the FF-PSO-PID algorithm can work effectively under complex signals. The effectiveness and superiority of the FF-PSO-PID algorithm is verified. It can effectively solve the problems of time lag, large error, and slow response of real-time hybrid tests (RTH).

## Figures and Tables

**Table 1:** Main parameters of the electro-hydraulic servo system model.

Parameters	Value
$\mathrm{Servo}\;\mathrm{amplifier}\;K_{a}$	0.004
$\mathrm{Feedback}\;\mathrm{amplifier}\;K_{s}$	90.9
Piston rod inner diameter	63 mm
Hydraulic cylinder internal volume	101 mm
Work itinerary	220 mm
$\mathrm{Effective}\;\mathrm{area}\;\mathrm{of}\;\mathrm{hydraulic}\;\mathrm{cylinder}\;A_{p}$	$0.00489\;m^{2}$
$\mathrm{Hydraulic}\;\mathrm{inherent}\;\mathrm{frequency}\;\omega_{r}$	314 rad/s
$\mathrm{Hydraulic}\;\mathrm{damping}\;\mathrm{ratio}\;\xi_{r}$	0.2
Servo valve rated current	40 mA
$\mathrm{Effective}\;\mathrm{bulk}\;\mathrm{modulus}\;\mathrm{of}\;\mathrm{elasticity}\;\beta_{e}$	$7\times 10^{8}\;N/m^{2}$
$\mathrm{Total}\;\mathrm{flow}\;\mathrm{pressure}\;\mathrm{coefficient}\;V_{t}$	$0.0010758\;m^{3}$
$\mathrm{Servo}\;\mathrm{valve}\;\mathrm{damping}\;\mathrm{ratio}\;\xi_{n}$	0.7
$\mathrm{Servo}\;\mathrm{valve}\;\mathrm{inherent}\;\mathrm{frequency}\;\omega_{n}$	753.6 rad/s
$\mathrm{Servo}\;\mathrm{valve}\;\mathrm{flow}\;\mathrm{gain}\;K_{sv}$	0.021 m/A

**Table 2:** Parameters of the PSO algorithm.

Parameters	Value
$\mathrm{Learning}\;\mathrm{factor}\;C_{1}$	2
$\mathrm{Learning}\;\mathrm{factor}\;C_{2}$	2
Inertia coefficientω	0.6
Population size m	100
Number of iterations K	30
Particle dimension D	3

**Table 3:** Conventional PID parameters and PSO-PID parameters.

Parameters	PID	PSO-PID
$K_{p}$	18	19
$K_{i}$	1	0.1498
$K_{d}$	0.1	0.0001

**Table 4:** Performance evaluation index results for conventional PID and PSO-PID.

Parameters	PID	PSO-PID
Final value C(∞)	110.224 mm	110.039 mm
$\mathrm{Rise}\;\mathrm{time}\;t_{r}$	0.007 s	0.006 s
$\mathrm{Peak}\;\mathrm{time}\;t_{p}$	0.46 s	0.36 s
Overshoot σ%	0.2%	0.036%

## Data Availability

Not applicable.
